# Propagation of Recombinant Genes through Complex Microbiomes with Synthetic Mini-RP4 Plasmid Vectors

**DOI:** 10.34133/2022/9850305

**Published:** 2022-08-02

**Authors:** Tomás Aparicio, Jillian Silbert, Sherezade Cepeda, Víctor de Lorenzo

**Affiliations:** Systems and Synthetic Biology Department, Centro Nacional de Biotecnología (CNB-CSIC), Campus de Cantoblanco Madrid 28049Spain

## Abstract

The promiscuous conjugation machinery of the Gram-negative plasmid RP4 has been reassembled in a minimized, highly transmissible vector for propagating genetically encoded traits through diverse types of naturally occurring microbial communities. To this end, the whole of the RP4-encoded transfer determinants (*tra*, *mob* genes, and origin of transfer *oriT*) was excised from their natural context, minimized, and recreated in the form of a streamlined DNA segment borne by an autoselective replicon. The resulting constructs (the pMATING series) could be self-transferred through a variety of prokaryotic and eukaryotic recipients employing such a rationally designed conjugal delivery device. Insertion of GFP reporter into pMATING exposed the value of this genetic tool for delivering heterologous genes to both specific mating partners and complex consortia (e.g., plant/soil rhizosphere). The results accredited the effective and functional transfer of the recombinant plasmids to a diversity of hosts. Yet the inspection of factors that limit interspecies DNA transfer in such scenarios uncovered type VI secretion systems as one of the factual barriers that check otherwise high conjugal frequencies of tested RP4 derivatives. We argue that the hereby presented programming of hyperpromiscuous gene transfer can become a phenomenal asset for the propagation of beneficial traits through various scales of the environmental microbiome.

## 1. Introduction

Microbiome engineering is one of the most promising fields of application of synthetic biology in areas as diverse as human therapeutics, crop improvement, and environmental bioremediation [1–3]. Communities can be designed from either scratch or the composition and functions of those already in place modified upon the introduction of one or more members into the existing partnership [4]. Alas, rational modification of a standing microbial network is often limited by the well-known phenomenon of colonization resistance (CR), i.e., the ability of well-balanced communities to prevent or inhibit the establishment of foreign members [5]. This can be due to a variety of factors, including physical barriers, production of inhibitory compounds, injection of toxins, metabolic incompatibility, and others [6–8]. An alternative to adding new partners to an existing group is the delivery and spreading of the DNA encoding the functions of interest through the standing assembly, in such a way that the whole may acquire novel traits without necessarily changing the earlier structure or composition [6, 9]. The technical challenge, in this case, boils down to developing effective systems for the propagation of engineered DNA through a complex microbial community.

Natural processes of horizontal gene transfer (HGT) include conjugation, transformation, and transduction as well as a growing number of intermediate mechanisms [10]. While all of them have been proven to drive the spreading of DNA at many different scales, conjugation looks like the process more amenable to programming in the short run for the sake of disseminating new genes/functions in a microbiome. Out of the whole collection of conjugal systems available, the one borne by plasmid RP4 (also called RK2) stands out as the basis for engineering robust gene spreading strategies. The RP4 plasmid was first discovered in 1969 as the agent responsible of antibiotic-resistant infections driven by *Klebsiella* and *Pseudomonas* sp. [11]. Since then, it has become a model of unrestrained bacterial conjugation and a source of DNA parts for genetic tools. RP4 has proven to be a superpromiscuous plasmid in terms of replication and conjugation. It is able not only to proliferate in all Gram-negative bacteria tested so far [12] but also to mediate conjugative self-transfer between virtually any member of this bacterial domain [13, 14]. The promiscuity of the conjugation machinery of RP4 also allows DNA transfer to Gram-positive bacteria [15], yeast [16, 17], and even mammalian cells [18]. Different segments of the plasmid have been incorporated into broad host range vectors frequently used to manipulate Gram-negative microorganisms, especially environmental bacteria. Given the efficacy of its conjugal transfer machinery, it is no surprise that segments of RP4 encoding transfer functions *mob* and *tra* have long been reused for mobilization of plasmids endowed with an *oriT* origin of conjugation among different types of bacteria [14, 19]. In a subsequent development, the whole RP4 plasmid or selected portions of it have been played on for delivering toxins in models of *Vibrio* infection in zebrafish and artemia [20], tagging endophytic bacteria of poplar plants with GFP [21], probing conjugation of *Pseudomonas* in microbial communities of domestic conduits [22] and sand filters [23], invading mouse gut community with libraries of plasmids and transposon vectors [24], delivering genes to bacteria in activated sludge [25], and isolating fastidious gut bacteria through metaparental mating [26]. Besides accrediting the highly promiscuous status of the RP4 transfer machinery, the wealth of data derived from these studies exposed that gene transfer in a community is largely run by a very active subpopulation of the whole, dominated by Pseudomonadales, Enterobacteriales, Burkholderiales, and Rhizobiales [22]. Also, available data indicate that most of the transfer events happen at the early stage of exposure of the group to the spreader, followed by the decay of the primary propagator and eventual stabilization of RP4 in the community [27].

Given the need of tools for rational fortification of microbiomes mentioned above and the phenomenal potential of RP4 to spread across a wide variety of microorganisms, we have exploited the functional parts of its conjugal transfer machinery and refactored them in the shape of a standardized self-transmissible plasmid vector. The result was a coherent toolset (pMATING platform) optimized for the delivery and circulation of recombinant genes in natural microbial consortia. The range of possible recipients was also explored by assembling pMATING derivatives endowed with origins of replication known to be functional in Gram-negative, Gram-positive, or yeast hosts. Furthermore, vectors were added with a broad host range toxin-antitoxin countersegregation device to secure a stable inheritance of the synthetic constructs in the absence of antibiotic selection. Finally, some factual impediments to conjugation (in particular, type VI secretion systems of the donors) were identified and removed as required. We advocate the pMATING vector to be a prototype of the genetic tool specifically devised for programming microbiome performance for a range of applications in human/animal health, agriculture, and the environment [28].

## 2. Results and Discussion

### 2.1. Reassembling Functional Parts of the PR4 Plasmid into Minimized Self-Transmissible Vectors

**Figure 1:**
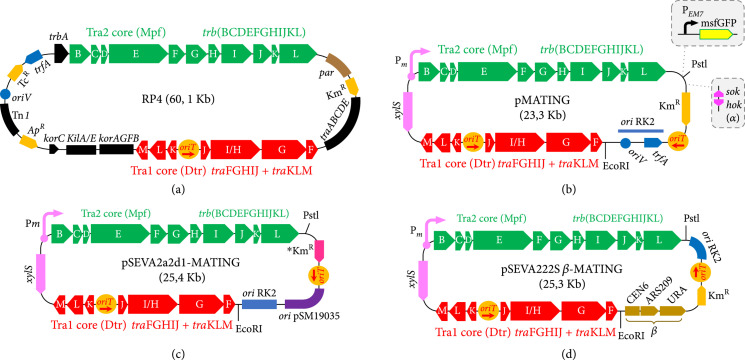
Schemes of plasmids used in this work. (a) The main gene features of the RP4 plasmid are depicted. Tra1 (DNA transfer region (Dtr)) and Tra2 (mating pair formation (Mpf)) gene clusters are highlighted in red and green, respectively [29]. (b) pMATING structure is shown: the MATING module is flanked by unique EcoRI/PstI sites, and the vector backbone displays a RK2 *ori*, the standard Km^R^ gene of SEVA vectors, and the *oriT* sequence. The *α* gadget (toxin-antitoxin system *hok-sok*) and the cassette P*_EM7_*-msfGFP are also shown as part of the derivatives pMATING*α* and pMATING*α*-msfGFP. (c) pSEVA2a2d1-MATING structure is depicted. The MATING module is loaded in a shuttle vector backbone containing a dual replicon RK2 (functional in Gram^-^ cells) and pSM19035 (functional in Gram^+^ cells), the *oriT*, and the Km^R^ gene of the *Staphylococcus aureus* pUB110 plasmid under the P*_veg_* promoter (conferring resistance to Km both in Gram^+^ and Gram^-^ bacteria). (d) Structure of pSEVA222S*β*-MATING: the MATING module is loaded in the pSEVA222S*β* backbone containing the RK2 *ori*, the *oriT* sequence, the standard Km^R^ gene of SEVA vectors, and the gadget *β*. The gadget includes the sequences for segregation (CEN6), replication (ARS209), and selection (URA) in *S. cerevisiae*.

The starting point of this work is a 19.5 kb synthetic DNA segment (that we call thereafter the MATING module) encoding an edited, streamlined, and compressed conjugation machinery of RP4 (Figure 1(a); [29]) including clusters Tra1 (DNA transfer region (Dtr)) and Tra2 (mating pair formation (Mpf)). The whole was designed with flanking EcoRI/PstI restriction sites for easing its reuse in a suite of replicons with the expectation that it could by itself promote the conjugal transfer of any DNA associated with it. To test this, the MATING segment was loaded into several vector backbones to assess the performance and host range of the resulting RP4 miniderivatives (Figure 1). First, the module was cloned in a pSEVA221, a vector containing the broad host range origin of replication RK2 and a Km^R^ cassette, to generate the reference plasmid pMATING (Figure 1(b)). Since RK2 *ori* derives from the origin of replication of RK2 [30], a plasmid virtually identical to RP4, this construct can be considered a minimized version of the parental plasmid with the potential to replicate and self-transfer to virtually any Gram-negative bacterium. The broad host range toxin-antitoxin *hok-sok* cassette (gadget *α* in the SEVA platform [31]) was added to this frame to produce pMATING*α* (Figure 1(b)). *hok-sok* stabilizes the plasmid in the host cells in the absence of antibiotic selection [32], a desirable property during *in vivo* experiments where selective pressure for plasmid maintenance is not feasible by antibiotic addition (see below). A further derivative (pMATING*α*-msfGFP; Figure 1(b)) was then built which was inserted with the monomeric superfolder GFP gene expressed through the constitutive promoter P*_EM7_* [33] for monitoring plasmid self-transfer events in environmental samples. A second plasmid variant was constructed for testing conjugation towards Gram-positive bacteria. In this case, the MATING segment was implanted as before in vector pSEVA2a2d1 (Supplementary Table S1). This is a standardized vector bearing a broad host range *oriV* RK2 for Gram-negative bacteria along with an unrestrained *oriV* of plasmid pSM19035 of *Streptococcus pyogenes* [34] for Gram-positive hosts. It also carries the Km^R^ gene *aadD* of the *S. aureus* pUB110 plasmid [35] expressed through the *P_veg_* promoter [36] which confers resistance to Km in both Gram-negative and Gram-positive bacteria. This resulted in plasmid pSEVA2a2d1-MATING (Figure 1(c); Supplementary Table S1). Finally, a separate plasmid to test conjugation towards *Saccharomyces cerevisiae* was constructed by inserting the MATING module in pSEVA222S*β* [29]. This is a standardized yeast shuttle vector not only endowed with an *oriV* RK2 and a Km^R^ gene but also added with a gadget (*β* in the SEVA nomenclature) with DNA sequences for replication (ARS209), segregation (CEN6), and selection (URA) in a URA-minus yeast. Appending the MATING module to this backbone rendered pSEVA222S*β*-MATING, a plasmid able to replicate in both *S. cerevisiae* and Gram-negative bacteria and thus potentially able to promote autoconjugative transfer towards budding yeast (Figure 1(d)). With these plasmids in hand, we then set out to investigate their actual value as a platform for the delivery of recombinant genes to other microbial receivers.

### 2.2. Conjugation Performance of pMATING between Gram-Negative Partners

**Figure 2:**
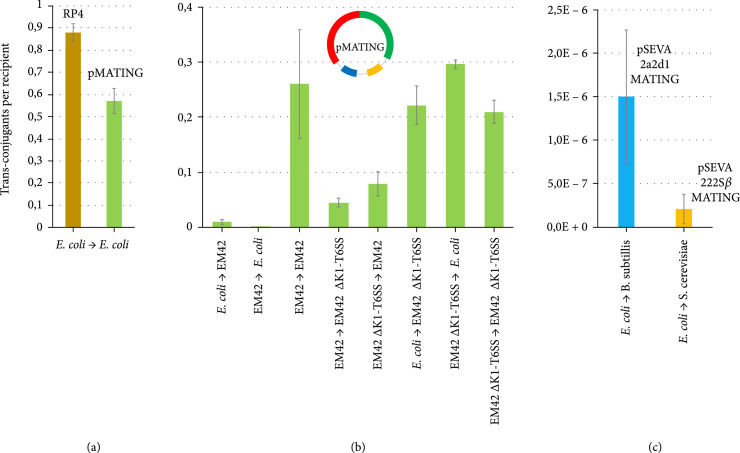
Conjugation transfer assays of mini-RP4 derivatives. (a) The performance of pMATING and RP4 transfer between *E. coli/E. coli* strains is shown. The efficiency of conjugational transfer was calculated as the ratio of transconjugants per recipient in a biparental mating assay. Straight arrows indicate the direction of transfer between a donor strain harbouring pMATING (arrow tail) and a recipient strain devoid of plasmid (arrowhead) in a given assay. (b) The performance of pMATING transfer between *E. coli/P. putida* and *P. putida/P. putida* strains is depicted with transfer efficiencies calculated as in (a). (c) Performance of transfer of pMATING derivatives from *E. coli* to *B. subtilis* (as a representative strain of the Gram^+^ group, pSEVA2a2d1-MATING) and from *E. coli* to *S. cerevisiae* (transkingdom conjugation, pSEVA222S*β*-MATING). Transfer efficiencies were calculated as in (a). Presented values are the mean of two independent replicas of each experiment. Detailed information including bacterial strains, mating temperature, mating media, and selective media for each experiment can be found in Table 1.

In the first series of experiments, biparental matings were set up to assay the ability of the RP4 miniderivatives described above to conjugally self-transfer among *E. coli* and the soil dweller *Pseudomonas putida* EM42 (a streamlined derivative of *P. putida* KT2440 [37]) and their combinations thereof. Matings were set up in solid media [38] by just mixing a donor harbouring the plasmid under scrutiny with receptor strains as detailed in Materials and Methods and Table 1. Mating efficiencies were grossly calculated as the number of transconjugants per recipient cell. To have a baseline for the ensuing experiments, the performance of pMATING was first tested when both donors and recipients were *E. coli* strains and compared with the transfer efficiency of the parental plasmid RP4 (Figure 2(a)). RP4 showed a transfer efficiency of 0.88 (88 transconjugants per 100 recipients) while pMATING manifested a comparable transfer rate of 0.57 (57 transconjugants per 100 recipients). When similar experiments were conducted between both *P. putida* donors and recipients, transfer frequencies were also high (0.26, Figure 2(b)). The lower percentage with respect to the all *E. coli* mating might stem from intrinsic differences between both bacteria but could also reflect the slower growth rate of the *P. putida pyrF* mutants used as donor strains [39]. Negative controls using donor strains of either *E. coli* or *P. putida* harbouring pSEVA221 without the MATING segment did not yield a detectable level of plasmid transfer.

**Table 1:** Donor and recipient strains, mating media and temperatures, and selective media used in biparental conjugation assays.

Mating strains		Mating media/Tª	Selective media	
Donor	Recipient		Transconjugants	Recipients
*E. coli* MC1061 DIAL EI *ΔdapA*-Eps-*Δ*Km^R^/pMATING	*E. coli* CC118	LB-DAP/37°C	LB-Km	LB
*E. coli* MC1061 DIAL EI *ΔdapA*-Eps-*Δ*Km^R^/RP4	*E. coli* CC118	LB-DAP/37°C	LB-Km	LB
*E. coli* MC1061 DIAL EI *ΔdapA*-Eps-*Δ*Km^R^/pMATING2*α*	*E. coli* CC118	LB-DAP/37°C	LB-Km	LB
*E. coli* MC1061 DIAL EI *ΔdapA*-Eps-*Δ*Km^R^/pSEVA221	*E. coli* CC118	LB-DAP/30°C	LB-Km	LB
*E. coli* CC118/pMATING	*P. putida* EM42	LB/30°C	M9-citrate-Km	M9-citrate
*E. coli* CC118/pMATING	*P. putida* EM42 *Δ*K1-T6SS	LB/30°C	M9-citrate-Km	M9-citrate
*P. putida* EM42 *ΔpyrF*/pMATING	*E. coli* CC118	LB/30°C	LB-Rif-Km	LB-Rif
*P. putida* EM42 *ΔpyrF Δ*K1-T6SS/pMATING	*E. coli* CC118	LB/30°C	LB-Rif-Km	LB-Rif
*P. putida* EM42 *ΔpyrF*/pSEVA221	*E. coli* CC118	LB/30°C	LB-Rif-Km	LB-Rif
*P. putida* EM42 *ΔpyrF*/pMATING	*P. putida* EM42	LB/30°C	M9-citrate-Km	M9-citrate
*P. putida* EM42 *ΔpyrF Δ*K1-T6SS/pMATING	*P. putida* EM42	LB/30°C	M9-citrate-Km	M9-citrate
*P. putida* EM42 *ΔpyrF*/pMATING	*P. putida* EM42 *Δ*K1-T6SS	LB/30°C	M9-citrate-Km	M9-citrate
*P. putida* EM42 *ΔpyrF Δ*K1-T6SS/pMATING	*P. putida* EM42 *Δ*K1-T6SS	LB/30°C	M9-citrate-Km	M9-citrate
*P. putida* EM42 *ΔpyrF*/pSEVA221	*P. putida* EM42	LB/30°C	M9-citrate-Km	M9-citrate
*E. coli* MC1061 DIAL EI *ΔdapA*-Eps-*Δ*Km^R^/pSEVA2a2d1-MATING	*B. subtilis* BG214	LB-DAP/30°C	LB-Km_5_	LB
*E. coli* MC1061 DIAL EI *ΔdapA*-Eps-*Δ*Km^R^/pSEVA222S*β*-MATING	*S. cerevisiae* CRY1-2	YPD-DAP-NaCl 0.5%	SC-Ura	YPD
*E. coli* MC1061 DIAL EI *ΔdapA*-Eps-*Δ*Km^R^/pSEVA222S*β*	*S. cerevisiae* CRY1-2	YPD-DAP-NaCl 0.5%	SC-Ura	YPD
*Novosphingobium* sp./pMATING*α*-msfGFP	*E. coli* CC118	LB/30°C	LB-Rif-Km	LB-Rif
*Pantoea* sp./pMATING*α*-msfGFP	*E. coli* CC118	LB/30°C	LB-Rif-Km	LB-Rif
*Buttiaxella* sp./pMATING*α*-msfGFP	*E. coli* CC118	LB/30°C	LB-Rif-Km	LB-Rif
*Aeromonas* sp./pMATING*α*-msfGFP	*E. coli* CC118	LB/30°C	LB-Rif-Km	LB-Rif
*Pseudomonas* sp-1./pMATING*α*-msfGFP	*E. coli* CC118	LB/30°C	LB-Rif-Km	LB-Rif
*Pseudomonas* sp-2./pMATING*α*-msfGFP	*E. coli* CC118	LB/30°C	LB-Rif-Km	LB-Rif

In the second round of tests, we inspected the ability of pMATING to move between different two Gram-negative species, i.e., from *E. coli* to *P. putida* and *vice versa.* As summarized in Figure 2(b), conjugation numbers for *E. coli-*to*-P. putida* matings were ~0.01, i.e., way lower than those seen earlier in *E. coli-*to*-E. coli* experiments. When *P. putida* was the donor, efficacy came still lower to 10^-4^, i.e., 1 transconjugant per 10^4^ recipients. We did however notice that the number of *E. coli* CFUs out of the mating mixtures with *P. putida* was considerably lower than expected (on average, 10^7^ viable cells *vs.* 10^8^ of *E. coli* to *E. coli* matings), suggesting that physical interspecies contact caused a degree of cell death in the population of the enterobacteria. How could this happen? *P. putida* KT2440 (and EM42, the derivative strain used in this work) is known to harbour active type VI secretion systems (T6SS), capable of killing neighbouring bacteria by injection of toxin cocktails upon cell-to-cell contact [40]. Since these systems have been shown to interfere with conjugation [41], we set out to inspect whether this was a factor for interspecies pMATING self-transfer.

### 2.3. T6SSs Check in the Conjugation Efficiency of Mini-RP4 Constructs

Out of the three T6SSs encoded in the genome of *P. putida*, the so-called K1-T6SS seems to be the most lethal in interspecies killing [40]. To examine whether this device was responsible for the poor transmission of pMATING when one of the conjugation partners was *P. putida*, a derivative of strain EM42 was deleted from the cognate gene cluster (see Materials and Methods) and used instead of the wild type in a separate series of intra/inter species matings. As summarized in Figure 2(b), when both donor and recipients were *P. putida* EM42 *Δ*K1-T6SS strains, transfer efficiencies between them were similar to those seen with parental partners (0.21). This figure came down to 0.04-0.07 when one *P. putida* partner was K1-T6SS^+^ and the other was ∆K1-T6SS, suggesting that the killing system influenced the process. The effect became more manifest in interpecies matings: *P. putida* EM42 *Δ*K1-T6SS transferred pMATING to *E. coli* at a 0.29 frequency, about the same rate when the donor was *E. coli* and the recipient was a T6SS-less *P. putida* strain (0.22). Elimination of K1-T6SS thus led to a sharp increase in transfer frequencies when *P. putida* was a partner of the mating mixture, whether as a donor or a recipient. This is not surprising because during conjugation cells became in intimate contact [42] which plausibly fires T6SS activity and toxin delivery to the recipients, followed by cell death. Intriguingly, the highest interference is observed when *P. putida* acts as the plasmid donor and *E. coli* is the receptor (Figure 2(b); 10^-4^). Since the mating efficiencies (as calculated in this article, Materials and Methods) reflect transconjugants/receptor ratios, this figure most likely exposes a massive death of *E. coli* receptors during pMATING transfer. Taken together, these results suggested that rather than acting as an asset to protect against invasion of DNA from occasional conjugal donors, T6SS performs as a way to prevent gene delivery to nonkin recipients. This issue—which is beyond the scope of this article—deserves further investigation.

### 2.4. Transfer of MATING-Bearing Plasmids to Gram-Positive Bacteria and Yeast

To further inspect the ability of the MATING module to autonomously mobilize associated DNA toward diverse acceptors, two additional series of conjugation tests were set up with *E. coli* as the donor and either *Bacillus subtilis* or *S. cerevisiae* as the receiving partners. In one case, the transferred construct at stake was pSEVA2a2d1-MATING, which can be selected and replicated in a suite of Gram-positive hosts (see above). The results (Figure 2(c)) indicated that the plasmid was successfully transferred from *E. coli* to *B. subtilis* BG214 although with low efficiency (~10^-6^ transconjugants *per* recipient cell) compared to the figures for matings among Gram-negative partners. Diagnostic PCR with primers L2-F1 and Tra1-R2 (Supplementary Table S2) were done on 5 isolates to verify the acquisition pSEVA2a2d1-MATING (Supplementary Figure S1). While this level of HGT is comparatively modest, it falls within the transfer efficiencies reported for the delivery of promiscuous plasmids from *E. coli* to Gram-positive species such as *Bacillus*, *Streptococcus, Lysteria*, *Lactobacillus*, *Staphylococcus*, and others [15, 43–45]. Finally, conjugation mixes were cast between *E. coli* donors bearing pSEVA222S*β*-MATING (Figure 1(c)) and yeast recipients under conditions comparable to the rest of the cases. In this experimental setup, *S. cerevisiae* cells received the recombinant construct at a frequency of $2\cdot 10^{-7}$ (Figure 2(c)). Five clones were analyzed by colony PCR with primers L2-F1 and Tra1-R2, demonstrating the presence of pSEVA222S*β*-MATING in yeast cells (Supplementary Figure S1). Negative controls with plasmid pSEVA222S*β* did not yield detectable plasmid transfer. This level of transfer is certainly discreet but indicates that the MATING segment can indeed foster transkingdom conjugation from *E. coli* to yeast, a phenomenon which has only been seldomly reported [16, 17, 46–48] and—to the best of our knowledge—never exploited further for biotechnological applications.

Although the analyses of conjugation described above were limited to just a few representative cases, the results accredited that the MATING module—at the very least—retains the conjugation capabilities of the RP4 parental plasmid in full whether the recipients are Gram-negative bacteria, Gram-positive species, or yeast. The next obvious question was whether this quality held also when the target of HGT was not a single microbial specimen but a complex natural community.

### 2.5. *P. putida* Propagates pMATING through Members of the Soil Microbiota

**Figure 3:**
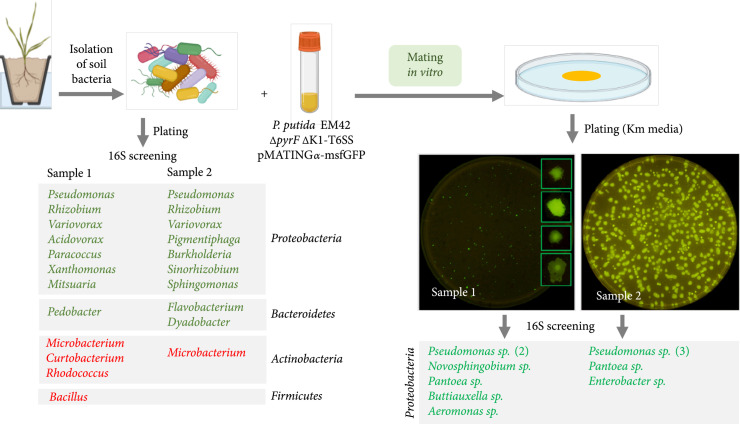
*P. putida* delivery of pMATING*α*-msfGFP to soil-extracted microbiota. The general workflow of the assay is shown: first, the soil bacterial population was isolated from two samples of soil and plated in nonselective media to analyze the culturable microorganisms. Bacterial colonies were characterized by 16S amplification and sequencing. Then, aliquots of soil bacteria were mixed with the donor strain *P. putida* EM42 *ΔpyrF Δ*K1-T6SS/pMATING*α*-msfGFP, and bacterial mixture was mated overnight in solid media. The bacterial patch was plated in selective media to isolate transconjugants: representative results of sample 1 (with the zoom-up of some colonies) and sample 2 are shown under UV light, exhibiting the GFP+ colonies. Fluorescent transconjugants were isolated and characterized by 16S amplification and sequencing, and the results can be seen under the pictures. Soil bacteria and transconjugants belonging to the Gram^-^ group are in green. Gram^+^ microorganisms appear in red.

To inspect the spreading potential of the pMATING frame across bacteria dwelling in environmental scenarios, we set up an experimental proxy in which the primary donor was *P. putida* and the recipients were the pool of species found in unprocessed soil samples from two sites in Spain (see Materials and Methods). The *Δ*K1-T6SS *ΔpyrF* variant of *P. putida* EM42 was chosen as the delivery strain for both avoiding liquidation of wild-type receptors-to-be in the environmental samples and also facilitating counterselection of donors after the mating experiments. Besides, the plasmid used in these experiments was pMATING*α*-msfGFP (Figure 1(b)) for easing visual identification of transconjugants (owing to the fluorescent marker) and increasing plasmid retention in transconjugants when antibiotic selection is not possible. Although the *oriV* of this MATING-containing plasmid mostly functions in Gram-negative bacteria (see above), we entertained that its behaviour could represent well the scope of the host range of the transfer system. To have also a tractable profile of potential recipients, the gross species composition of the culturable fraction of the target soils was determined. To this end, portions of the samples were suspended and diluted in PBS buffer 1X, plated in MT-Cx media, and incubated for 3 days at 30°C (Materials and Methods). 16S sequences of the grown colonies (Figure 3) revealed the presence of a variety of Gram-negative and Gram-positive genera mostly belonging to the phyla Proteobacteria, Actinobacteria, Bacteroidetes, and Firmicutes. As a control, aliquots of the same suspensions were plated directly on MT-Km-Cx agar plates to expose the background of Km^R^ bacteria in the environmental samples which, as expected [49], turned out to be large (~10% of all culturable bacteria in soil, not shown).

Once the partners of the conjugation assays were settled, aliquots of the soil-extracted bacterial pool were mixed with the donor strain *P. putida Δ*K1 *ΔpyrF* (pMATING*α*-msfGFP). The samples were concentrated, laid on top of MT-Cx agar plates, and grown overnight at 30°C (Materials and Methods). The bacterial mass was then resuspended, spread on MT-Km-Cx plates, and incubated at 30°C for 3 days. Controls included mixtures of the same soil samples with strain *P. putida Δ*K1 *ΔpyrF* (pSEVA221*α*-msfGFP), which harbours the same vector backbone but is devoid of the conjugative module. As expected, an abundant background of nonfluorescent Km^R^ colonies grew on the plates of both test and control mating mixtures. However, fluorescent colonies were not spotted in any of the negative controls (not shown). In contrast, GFP^+^ clones could be found out of mixtures originating in soil samples S1 and S2 subject to mating with the donor bearing pMATING*α*-msfGFP (Figure 3). 30 fluorescent transconjugants were exhaustively reisolated in MT-Km-Cx medium and subjected to diagnostic PCR with primers L2-F1 and Tra1-R2 (Supplementary Table S2) to verify acquisition and retention of pMATING*α*-msfGFP. All tested isolates of this sort were PCR-positive for the MATING module (Supplementary Figure S1), proving the transfer of the plasmid to these members of the soil samples. Subsequent amplification and determination of 16S sequences enabled identification of the thereby isolated recipients. These included several families of Proteobacteria, i.e., strains of the genus *Pseudomonas* (*Pseudomonadaceae*, 48%), followed by members of the genus *Novosphingobium* (*Sphingomonadaceae*, 19%), *Pantoea* (*Erwiniaceae*, 16%), *Buttiauxella* (*Enterobacteriaceae*, 9%), and *Aeromonas* (*Aeromonadaceae*, 6%) in S1 and *Pseudomonas* (75%), *Enterobacter* (*Enterobacteriaceae*, 16%), and *Pantoea* (*Erwiniaceae*, 8%) in S2.

While these observations demonstrated the ability of *P. putida* to conjugatively deliver, directly or indirectly, pMATING*α*-msfGFP to a wide variety of Gram-negative bacteria, note also that the figures presented are likely to be a major underestimation of the actual potential of the engineered transfer device. First, the donor strain bears several genomic deletions (not only *pyrF* but also various others of strain EM42 in respect to the parental *P. putida* KT2440 [37]) that are likely to compromise environmental fitness in competition with others. Second, only a small fraction of soil bacteria are culturable [50]. Finally, the fact that some common dwellers of soil microbiota (*Rhizobium*, *Variovorax*, *Xhantomonas*, etc.) were not receptive to plasmid transfer could be due to the efficacy of innate mechanisms to prevent exogenous DNA acquisition, i.e., T6SS interference, cell wall composition, restriction/modification systems, etc. In contrast, species less represented in the soil microbiota (e.g., *Pantoea*, *Novosphingobium* or *Aeromonas*) showed up as frequent receptors of MATING-mediated conjugative transfer. This may reflect the phenomenon reported by [51] on the presence in microbial communities of a super-HGT proficient subpopulation that is different in numbers and species from the overall configuration of the whole set.

### 2.6. In Situ Delivery pMATINGα-msfGFP to a Soil Community

**Figure 4:**
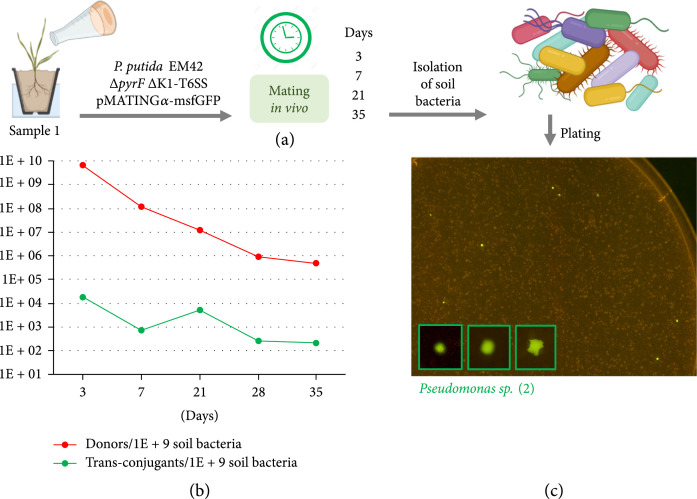
*P. putida* delivery of pMATING*α*-msfGFP to soil bacteria microbiota *in vivo.* (a) General workflow of the *in vivo* assay: a natural sample of soil and ground cover was inoculated with the donor strain *P. putida* EM42 *ΔpyrF Δ*K1-T6SS/pMATING*α*-msfGFP and incubated 35 days outdoors. Soil bacteria were sampled at different time points and plated on different media. (b) Soil bacteria, donors, and transconjugants were enumerated on, respectively, MT-Cx, MT-Cx-Km-Ura, and MT-Cx-Km (only fluorescent colonies). Number of donors and transconjugants was normalized to 10^9^ soil bacteria, and results were plotted for each sampling day. (c) Detail of a plate showing GFP+ trans-conjugants (day 7) and zoom-up of three fluorescent colonies.

The ensuing question on the value of the MATING module to promote HGT was whether the conjugation scheme depicted above could apply to bacteria residing in undisturbed environmental scenarios. To gain an insight into this question, simple microcosms were set up in which a patch of the upper soil and cover of sample 1 was placed in a plant pot and a culture of the donor strain *P. putida Δ*K1 *ΔpyrF*/(pMATING*α*-msfGFP) was inoculated on top of it (Figure 4(a)). As before, a control experiment was arranged in the same conditions with *P. putida Δ*K1 *ΔpyrF* bearing the nontransmissible plasmid pSEVA221*α*-msfGFP. Both microcosms were maintained with daily watering in an open backyard in which temperatures varied within a 10-32°C range. Soil samples were taken on days 3, 7, 21, and 35 (Figure 4(a)) and plated on different media and cultured for 3 days at 30°C to enumerate (i) the total population of culturable bacteria (colonies growing on MT-Cx), (ii) the whole of soil transconjugants (fluorescent colonies in MT-Km-Cx), and (iii) the number of *P. putida* donors (fluorescent colonies growing in MT-Km-Cx-Ura). As before, while no fluorescent colonies were observed in the negative control, GFP^+^ transconjugants were spotted along the whole sampling period when the *P. putida* donor carried pMATING*α*-msfGFP. The number of donors and transconjugants was normalized to the total number of culturable bacteria in the soil sample at each time point. The results (Figure 4(b)) exposed various informative features. First, there was a sharp decline in the numbers of *P. putida* donors along time (~4 logs in 35 days). This was not unexpected, not only because the donor strain surely has a lower environmental fitness (see above) but also because inoculation of soil with high numbers of bacteria creates a niche for grazing protists [52]. Second, the absolute maximum number of transconjugants was observed soon after donor inoculation (~10^4^ transconjugants/10^9^ soil bacteria on day 3), coming down to ~10^2^ transconjugants/10^9^ soil bacteria at the end of the experiment. Such transfer kinetics is comparable to that reported by Fan et al. [27] for spreading the actual RP4 from *P. putida* to a bacterial soil community—thereby suggesting that the transfer potential of the naturally occurring plasmid is fully preserved in the streamlined vector. Finally, an inspection of the 16S sequence of the transconjugants revealed two strains of resident *Pseudomonas* (in fact the same identified in the experiments of Figure 3) as the main receptors of conjugative transfer (Figure 4(c)). Although (as before) we deem the results of Figure 4 to underestimate the real transfer rates of pMATING, they also show that plasmid delivery occurs at significant levels when an engineered donor is put in contact with an undisturbed complex microbiota.

### 2.7. Environmental Bacteria Become Conjugation Proficient upon Acquisition of pMATING

**Figure 5:**
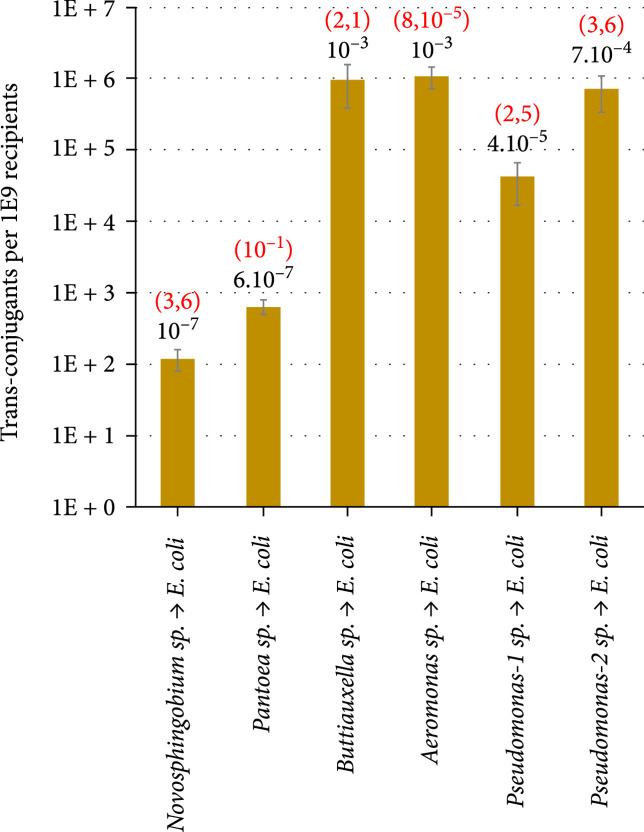
Back transfer of pMATING*α*-msfGFP from wild-type soil bacteria to *E. coli*. Biparental mating assays between soil isolates harbouring pMATING*α*-msfGFP (donors) and *E. coli* CC118 (receptor) showed conjugational transfer in all cases. Transfer efficiencies (numbers in black) were calculated as explained in Figure 2(a) but were plotted in logarithmic scale as transconjugants per 10^9^ recipients for a better visualization. Recipient survival was also quantified using the Receptor Survival Rate (RSR, numbers in red) to asses *E. coli* cell death during mating experiments. RSR≥1 reflects no impact on receptor survival, and RSR<1 shows a decrease on receptor survival (see text for details). Presented values are the mean of two independent replicas of each experiment. Detailed information including bacterial strains, mating temperature, mating media, and selective media for each experiment can be found in Table 1.

The remaining question on the HTG vector described above is whether those recipients which have acquired the synthetic plasmid become proficient as donors in subsequent rounds of conjugation with other bacteria. The possibility of secondary transmissions was tested by setting separate matings between environmental transconjugants bearing pMATING*α*-msfGFP isolated in the experiments already described and *E. coli* CC118 cells as possible recipients. To this end, 6 clones of *Butiauxella*, *Pseudomonas*, *Aeromonas*, *Novosphingobium*, and *Pantoea* were mated with the destination *E. coli* strain, and conjugative transfer was analyzed as before, i.e., determining the ratio *E. coli* transconjugants/*E. coli* recipients. Figure 5 shows that all tested strains were able to transfer the plasmid, albeit with different efficiencies. *Butiauxella* sp. and two species of *Pseudomonas* achieved the highest levels of back transfer of pMATING*α*-msfGFP (10^-3^, $7\cdot 10^{-4}$, and $4\cdot 10^{-5}$, respectively), while *Novosphingobium* sp. and *Pantoea* sp. showed a lower range (10^-7^). Yet, when *Aeromonas* was used as the donor, the results were somewhat paradoxical and indicative of an additional player in the mating progression. As shown in Figure 5, the transfer efficacy was comparatively very high (10^-3^), but the net number of transconjugants and the whole of the recipient *E. coli* population that surfaced from the mating process were very low. As this could be due to the massive killing of *E. coli* by *Aeromonas* upon direct cell-to-cell contact, we quantified in all cases what we called Receptor Survival Rate (RSR) as a measure of the recipient cell death during conjugation experiments. For this, the mean number of *E. coli* CFUs after mating with a given donor strain was divided by the mean number of recipients in a separate experiment of reference where no cell death was expected, e.g., *E. coli* to *E. coli* matings reported above (Figure 2(a)). Under this frame, RSR values close to 1 mean no cell death, while figures<1 are indicative of lower rates of receptor survival and potential killing by the donor. As shown in Figure 5, donor *Pantoea* sp. had a strong effect on recipient survival, i.e., RSR~10^-1^ (one out of ten receptors survives the contact), but it also displays a poor performance as a conjugative donor (10^-7^). In contrast, *Novosphingobium* sp. did not affect *E. coli* survival (RSR 3.6), but the conjugal transfer was very low (10^-7^). However, *Aeromonas* sp. seems to kill *E. coli* massively, i.e., RSR $\sim 8\cdot 10^{-5}$ (only one out of 10^5^ recipients stays alive), while keeping the transfer rate surprisingly high (10^-3^). Finally, two *Pseudomonas* strains and *Buttiauxella* sp. did not show any impact on *E. coli* survival and transfer rates varied between 10^-3^ and 10^-5^. These data (Figure 5) leave two important pieces of information. One is that pMATING endows a range of environmental bacteria with the capacity to pass on the plasmid to nearby residents, albeit with very different frequencies. The other is that barriers to HGT do not reside exclusively in the recipients-to-be as a way to prevent the invasion of foreign DNA. It seems also that some potential donors may actively avoid giving their DNA away to nonkin partners by activating killing-upon-contact mechanisms towards some species. T6SS seems to be one of such mechanisms (see above), but there may be others still to be explored. In any case, these observations, along with the already documented asymmetry of plasmid acquisition in environmental populations [22], highlight the many players of conjugative dynamics in environmental communities and pave the way for developing orthogonal HGT devices which operate independently of such donor/receptor specifics. In the meantime, the data above enable us to sketch what a usable vector for propagating recombinant genes in an environmental microbiome could look like.

### 2.8. Conclusion

**Figure 6:**
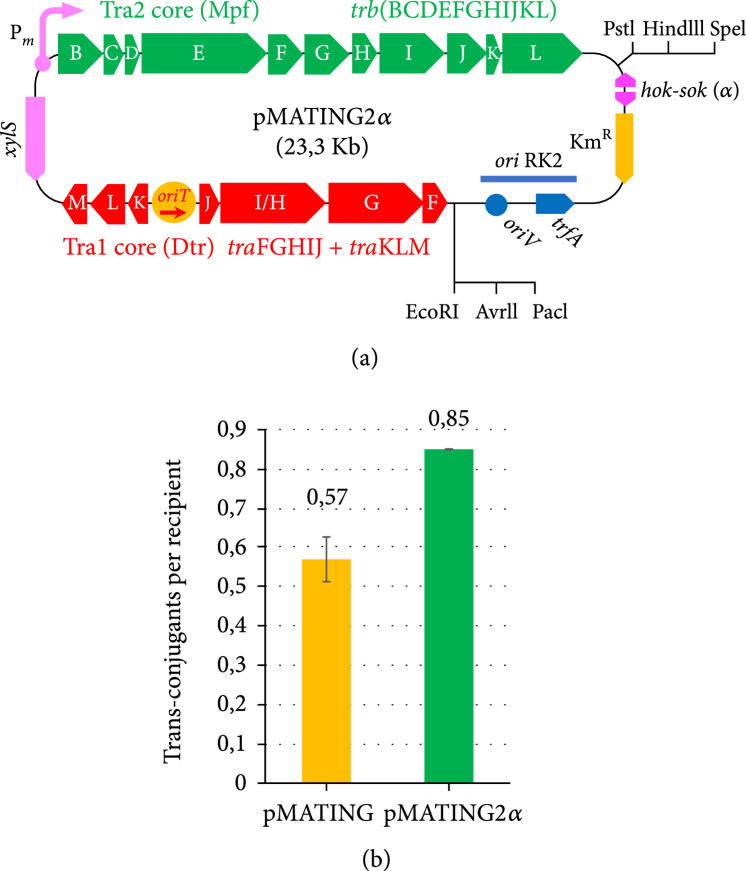
pMATING2*α* derivative displaying a single *oriT* sequence overperforms pMATING conjugational performance. (a) Plasmid structure of pMATING2*α*: the plasmid is identical to pMATING*α* but devoid of the *oriT* sequence present in the vector backbone. Unique restriction sites flanking the MATING module are depicted. (b) Biparental mating assays were run between *E. coli/*pMATING2*α* (donor) and an *E. coli* receptor. Results are compared with similar assays using pMATING, which harbours two *oriT* sequences (vector backbone and Tra1 region). Presented values are the mean of two independent replicas. Detailed information including bacterial strains, mating temperature, mating media, and selective media for each experiment can be found in Table 1.

Based on the above, we have developed the vector pMATING2*α* (Figure 6(a); GenBank accession number OM972019), which is an optimized version of the precursors utilized along this work. The structure of pMATING2*α* keeps the basic arrangement of pMATING with some significant additions. First, it is endowed with the *α* SEVA gadget (the *hok/sok* stabilization system used already in pMATING*α*-msfGFP) to secure plasmid maintenance in the absence of antibiotic selection. Also, unlike all the miniRP4 derivatives used in this work, pMATING2*α* has only one *oriT* sequence in its frame instead of the two: one in the MATING module as part of the Tra1 region and a second copy in the SEVA vector backbone. This redundancy (irrelevant for the short-time experiments shown above) may influence the conjugation performance due to double nicks at both *oriT*s by the relaxase, generating plasmid instability and/or DNA rearrangements in the long run. As shown in Figure 6(b), when pMATING2*α* (Figure 6(a)) was assayed in *E. coli/E. coli* matings, this edited construct overperformed the conjugational transfer ratio of pMATING by ~50%, reaching a 0.85 efficiency. This means that the vector keeps and even surpasses the conjugative ability of the precursor plasmid, reaching efficiencies of transfer almost identical to those reported for the original RP4 (0.88, Figure 2(a)). Finally, pMATING2*α* has 6 unique sites (EcoRI, AvrII, PacI, PstI, HindII, and SpeI) available for insertion of recombinant genes either through classical restriction cloning or Gibson assembly. Complete standardization of pMATING2*α* in a SEVA format [53] was not possible as many restriction sites present in the *tra* regions of the MATING segment were unfeasible to remove without loss of function.

While earlier works have reported conjugative transfer or RP4 to diverse microbial communities [22, 23, 27, 51], to the best of our knowledge, pMATING2*α* is the first vector specifically designed and optimized to deliver recombinant genes through environmental microbiomes. The construct size is not only ~1/3 of the parental plasmid, thereby simplifying its handling. But also, because of keeping part of the modularity of the SEVA architecture, the exchange of some of its functional modules (e.g., antibiotic markers and expression cargoes), according to needs, is straightforward. Finally, owing to its ability to turn recipients into secondary donors (see above and [25]), pMATING2*α* can become a phenomenal tool for the circulation of the genes of interest across complex bacterial communities. In sum, we argue that pMATING2*α* and vectors inspired in it thereof will be instrumental to fortify target bacterial niches for the sake of therapeutical, agricultural, or environmental applications.

## 3. Materials and Methods

### 3.1. Strains and Media

The strains used in this work are listed in Supplementary Table S1. *E. coli* and *P. putida* strains were routinely grown with shaking (170 rpm) in liquid LB [54] at 37°C and 30°C, respectively. *S. cerevisiae* was grown at 30°C in solid YPD (10 g L^-1^ yeast extract, 20 g L^-1^ peptone, 20 g L^-1^ dextrose, and 2% agar) or solid SC-Ura (6.7 g L^-1^ yeast nitrogen base w/o amino acids, 1.92 g L^-1^ yeast synthetic drop-out medium supplement without uracil, 20 g L^-1^ dextrose, and 2% agar). M9 minimal media was prepared according to [54] and supplemented with 0.2% citrate or glucose as carbon sources. 2.5 ml of Goodies Solution (300 mg L^-1^ HBO_3_, 50 mg L^-1^ ZnCl_2_, 30 mg L^-1^ MnCl_2_·4H_2_O, 200 mg L^-1^ CoCl_2_, 10 mg L^-1^ CuCl_2_·2H_2_O, 20 mg L^-1^ NiCl_2_·6H_2_O, 30 mg L^-1^ NaMoO_4_·2H_2_O, 2.5 g L^-1^ FeSO4·7H_2_O, and 61.62 g L^-1^ MgSO4·7H_2_0) was added per liter of M9 agar media when necessary. Cycloheximide (Cx) was added to the media when stated at 100 *μ*g ml^-1^ to inhibit the growth of fungi. Solid media was prepared by adding 2% agar to liquid media. MT media corresponds to liquid M9-glucose-goodies. Antibiotics were included in the growth media at the following concentrations: 50 *μ*g ml^-1^ of kanamycin (Km) for *E. coli*, *P. putida*, and environmental samples, while 5 *μ*g ml^-1^ of the same antibiotic was used for *B. subtilis*; 30 *μ*g ml^-1^ of chloramphenicol (Cm) for *E. coli*; 50 *μ*g ml^-1^ of streptomycin (Sm) for *E. coli* and 100 *μ*g ml^-1^ of Sm for *P. putida*; and 50 *μ*g ml^-1^ of rifampicin (Rif) and 10 *μ*g ml^-1^ of tetracycline (Tc). 2,6-Diaminopimelic acid (DAP) was added to the media at 20 *μ*g ml^-1^ to sustain the growth of the DAP auxotrophic strains of *E. coli* MC1061 DIAL EI *Δ*dapA, and 20 *μ*g ml^-1^ of uracil (Ura) was added to sustain the growth of *ΔpyrF* strains of *P. putida*. PBS buffer pH 7.4 10X contains 80 g L^-1^ NaCl, 2 g L^-1^ KCl, 14.4 g L^-1^ Na_2_HPO_4_, and 2.4 g L^-1^ KH_2_PO_4_.

### 3.2. General Procedures and Primers

DNA manipulations were carried out using routine laboratory techniques [54] and manufacturer recommendations. Plasmid purifications were performed with the QIAprep® Spin Miniprep Kit (Qiagen, Valencia, USA). Primers were obtained from Sigma-Aldrich (St. Louis, USA) and are listed in Supplementary Table S2. PCR amplifications used for plasmid construction were performed with Q5 polymerase (New England Biolabs, Ipswich, USA) while diagnosis PCRs were conducted with DNA Amplitools Master Mix (Biotools, Madrid, Spain). For *S. cerevisiae* diagnostic PCRs, one colony of yeast was resuspended in 50 *μ*L of solution 1 (Zymoprep™ Yeast Plasmid Miniprep II kit-ZymoResearch), and the resuspension was added with 20 U/ml zymolyase (ZymoResearch, REF #E1004). The sample was incubated at room temperature for 10 minutes and then centrifuged at 4000 rpm/10 seconds. The supernatant was removed, and the cell pellet was heated at 95°C for 5 minutes and then resuspended in 50 *μ*L H_2_O. 5 *μ*L of the sample was used as a template in a standard PCR reaction (25 *μ*L final volume). For isothermal assembly, Gibson Assembly® Master Mix (New England Biolabs, Ipswich, MA, USA) was used. USER® Cloning (New England Biolabs, Ipswich, MA, USA) was used for USER assembly. Colony PCR in *S. cerevisiae* was performed as described in [29]. The Nucleospin® Gel and PCR Clean-up Kit (Macherey-Nagel, Düren, Germany) were used for purification of PCR amplicons. DNA sequencing was performed in Macrogen-Spain. Transformation of *E. coli* laboratory strains was carried out with chemically competent cells using the CaCl_2_ method [54]. *P. putida* strains were transformed with plasmids via tripartite mating [55] and selected in solid M9 minimal media supplemented with 0.2% w/v citrate and appropriate antibiotics. Other plasmids were electroporated in *P. putida* as described in [56]. Other mating protocols used in this work are explained below.

### 3.3. Construction of Strains

The DAP auxotrophic strain *E. coli* MC1061 DIAL EI *Δ*dapA::FRT-Km^R^-FRT-Eps (unable to grow on LB unless 20 mg L^-1^ are added to the media) was a gift from Tom Ellis (Imperial College, London). The Km resistance cassette was removed by transforming the strain with plasmid pFLP2 (Supplementary Table 1) and plating the transformation on LB-DAP-Sucrose 5% solid media at 37°C. Thermal induction of the plasmid-encoded FLP recombinase (driven by the cI857-P_L_ expression system) and the counterselection of plasmid pFLP2 with sucrose allowed simultaneously eliminating the Km^R^ gene and curing the plasmid in one single step. Colonies were streaked in LB-DAP, LB-DAP-Km, and LB-DAP-Ap, and a Km^S^Ap^S^ colony was selected. The strain was restreaked in the same three media to confirm the phenotype, giving raise to *E. coli* MC1061 DIAL EI *Δ*dapA-Eps strain. To delete the type VI secretion system K1-T6SS in strains *P. putida* EM42 and *P. putida* EM42 *ΔpyrF*, the *I-SceI* deletion system was used [57]. Briefly, both strains were transformed with pSEVA412S-3104-3110 and cointegrates selected in LB-Sm. Upon transformation with pSEVA528S and selection on LB-Tc, expression of *I-SceI* endonuclease was induced with 1 mM 3-methyl-benzoate (3 MB) in liquid LB for 4 h. Cultures were plated in LB and colonies streaked in LB and LB-Sm. Sm^S^ clones were subjected to diagnostic PCR with oligo pairs PP3104-EcoRI-F/R (deletion: 1.0 Kb; wt: no amplification) and PP3106-F/R (deletion: no amplification; wt: 0.5 Kb) to identify the deleted strains *P. putida* EM42 *Δ*K1-T6SS and *P. putida* EM42 *ΔpyrF Δ*K1-T6SS.

### 3.4. Plasmid Construction

pMATING, pMATING*α*, and pSEVA222S*β*-MATING were constructed by excising the MATING module from pTRANS (Supplementary Table S1) with EcoRI/PstI and ligating the resulting 19.5 Kb fragment to, respectively, pSEVA221, pSEVA221*α*, and pSEVA222S*β* plasmids restricted with the same enzymes. To generate pMATING*α*-msfGFP, the P*_EM7_*-msfGFP cassette was amplified with primers Gibson-PstI-msfGFP-F/R (0.8 Kb) using pSEVA227-M-P*_EM7_*-msfGFP as a template. pMATING*α* was cut with PstI and Gibson assembled with the purified PCR fragment. In the resulting construct, one PstI site is mutated so that the MATING module plus the P*_EM7_*-msfGFP cassette is flanked by EcoRI/PstI sites. pSEVA221*α*-msfGFP was done in the same way except for amplifying the P_EM7_-msfGFP cassette with primers Gibson-PstI-msfGFP-F2/R and using pSEVA221*α* (PstI restricted) as the vector backbone. In this case, the final construct contains the cassette flanked by two PstI sites. To obtain pMATING2*α*, which displays a single origin of transfer, the *oriT* of the plasmid pSEVA221*α* was first deleted by amplifying the vector with primer pairs T1-Fw/Del-oriT-Rv (1.8 Kb fragment including Km^R^-*hok/sok*-MCS) and Del-oriT-Fw2/T1-Rv (2.3 Kb fragment, *oriV*_RK2_) and assembling the two purified PCR fragments by Gibson. Deletion of *oriT* in the resulting pSEVA221*α*^oriT^ was checked by sequencing with primers Km-check1 and trfA-check1. In a second step, pSEVA221*α*^oriT^ was cut with EcoRI/PstI and ligated with the MATING module excised from pTRANS with the same enzymes to produce pMATING2*α*. Finally, to assess the autoconjugative transfer between Gram-negative and Gram-positive bacteria, pSEVA2a2d1-MATING was constructed by ligating the MATING module into the pSEVA2a2d1 (Supplementary Table S1) restricted with EcoRI/PstI. All the clonings explained above were done by delivering ligations to chemically competent *E. coli* CC118 cells and selecting transformants on LB with appropriate antibiotics. Plasmid integrity was confirmed through restriction analyses and DNA sequencing whenever required.

### 3.5. Biparental Conjugation Assays

The experiments described here were aimed at quantifying the ability of mini-RP4 derivatives to self-transfer by conjugation between donor and recipient strains of different microorganisms. To counterselect donors after conjugation, *E. coli* donors with auxotrophy for DAP were used in matings *E. coli* → *E. coli* and *E. coli* → *B. subtilis*, whereas *P. putida* EM42 *ΔpyrF* donors (auxotrophs for uracil) were employed for matings *P. putida* → *P. putida*. For *P. putida* → *E. coli* matings, donors were counterselected upon Rif sensitivity of *P. putida*. The uracil auxotrophy was used to discriminate between *P. putida* donors and recipients upon the inability of *ΔpyrF* donors to grow on Ura^-^ minimal media. Note that even in rich media containing uracil (i.e. LB), *pyrF* mutants grew slower than wild-type bacteria [39] entering a bias in CFU counting following conjugation in LB.

For matings *E. coli* → *P. putida*, recipients were selected in minimal media with citrate as the sole carbon source, where *E. coli* strains cannot grow. Donor and recipient strains, mating temperatures, mating media, and selective media for recipients and transconjugants for each experiment can be found in Table 1. Biparental conjugation assays were done as follows: for experiments involving *E. coli* and *P. putida*, the donor strain harbouring the mini-RP4 derivative (or a control plasmid without the MATING module) and the recipient strain (devoid of plasmids) were grown overnight on 3 mL LB plus appropriate antibiotics to maintain the plasmid (Km_50_ for donors) or to select chromosomal markers (Rif for *E. coli* CC118). 20 *μ*g mL^-1^ of DAP was added to cultures of *E. coli* MC1061 DIAL EI *ΔdapA*-Eps-*Δ*KmR. After overnight growth, cultures were adjusted to $\text{O}{\text{D}}_{600}=1.0$ by centrifuging an aliquot, removing the supernatant, and resuspending the cellular pellet in 1 mL of 10 mM MgSO_4_. In order to eliminate antibiotics, 100 *μ*L of donor/recipient suspensions was mixed in one Eppendorf tube, 800 *μ*L of 10 mM MgSO_4_ was added, and the sample was centrifuged at 11000 rpm/2 min. After supernatant removal, the cellular pellet was resuspended in 20 *μ*L of 10 mM MgSO_4_, and the sample was carefully poured on top of a solid media plate, air-dried for 10 min, and incubated 18 h at the appropriate temperature to allow conjugation. An inoculation loop was then used to scrape out the cellular patch, and the sample was resuspended in 1 mL of 10 mM MgSO_4_ by gentle pipetting. Dilutions of samples were plated in selective media to quantify the number of recipients and transconjugants. To assay plasmid transfer between environmental strains harbouring pMATING*α*-msfGFP and *E. coli* CC118, the same procedure detailed above was applied. Previously, the rifampicin sensitivity of donor strains was checked in LB-Rif solid media: all tested strains were sensitive to rifampicin (data not shown), so the selection of transconjugants was done in LB-Km-Rif solid media. To calculate the Recipient Survival Rate (RSR), first the number of *E. coli* receptors after conjugation with a given wild-type strain was counted. To compare these figures with the expected number of receptors in conditions where donor/receptor interaction should not result in cell death, the mean number of receptors extracted from *E. coli*/*E. coli* mating experiments was calculated (3.9 E+8). RSR values are the result of dividing the mean *E. coli* receptors in a given mating assay with a wild-type donor harbouring pMATING*α*-msfGFP by 3.9 E+8. For experiments between *E. coli* MC1061 DIAL EI *ΔdapA*-Eps-*Δ*Km^R^/pSEVA2a2d1-MATING and *B. subtilis* BG214, the strains were grown at 30°C overnight in 3 mL LB-Km and LB, respectively. Cultures were inoculated in fresh media at OD_600_~0.1 and grown at 30°C until OD_600_~0.5. Then, cultures were adjusted to $\text{O}{\text{D}}_{600}=1.0$ in 10 mL of 10 mM MgSO_4_, and 5 mL of donor and recipient strains was mixed in 50 mL Falcon tubes and centrifuged at 4000 rpm/10 min. After removing the supernatant, the bacterial pellet was resuspended in 1 mL of the same solution. Cells were spun down at 11000 rpm/2 min and resuspended in 200 *μ*L of 10 mM MgSO4. The sample was placed on top of a solid media plate, air-dried for 10 min, and incubated 18 h at 30°C. Bacterial cells were recovered as explained above, and several dilutions were plated in selective media for recipients and transconjugants. For experiments between *E. coli* and *S. cerevisiae*, a suitable media to sustain the growth of both microorganisms was first tested. While *E. coli* was not able to thrive on the classical media for budding yeast cultivation (YPD, SC-Ura), YPD supplemented with NaCl 0.5% was shown to allow the growth of *E. coli* and was also appropriate for *S. cerevisiae* (data not shown). On this basis, the donor strain *E. coli* MC1061 DIAL EI *ΔdapA*-Eps-*Δ*Km^R^ harbouring pSEVA222S*β*-MATING or pSEVA222S*β* (control) was grown on 3 mL LB-DAP-Km at 37°C overnight and an aliquot of the culture adjusted to $\text{O}{\text{D}}_{600}=1.0$ in 10 mM MgSO^4^. *S. cerevisiae ura3* recipient strain (auxotroph for uracil) was streaked in YPD solid media and incubated 48 h at 30°C. Yeast colonies were resuspended in 10 mM MgSO_4_, and the sample was adjusted to OD_600_ ~10.0. Mating samples were set up by mixing 0.8 *μ*L of *E. coli* and 10 *μ*L of *S. cerevisiae* strains. This resulted, approximately, in a cellular ratio donor : recipient of 1 : 12.5. The ~11 *μ*L samples were placed on top of a YPD-DAP-NaCl 0.5% plate and incubated at 30°C for 18 h. As before, the cellular patches were scraped out and resuspended in 1 mL 10 mM MgSO_4_ and several dilutions plated either on YPD (to count recipients) or in SC-Ura solid media (to count *S. cerevisiae* transconjugants expressing the URA gene from the plasmid). Transconjugants of *E. coli*, *P. putida*, *B. subtilis*, and *S. cerevisiae* obtained in the experiments described above were analyzed by PCR to further check the presence of the mini-RP4 derivative in the receptor cells. Five to ten colonies of each set of experiments were amplified by PCR with primers L2-F1/Tra1-R2. All tested colonies showed the expected band of 0.5 Kb corresponding to the amplification segment between the *xylS* and Tra1 region of the MATING module (Supplementary Figure S1). The efficiency of conjugal transfer was calculated by counting the transconjugants and the recipients after the mating procedure and dividing both figures to obtain the ratio transconjugants/recipients. Two independent biological replicates were done for all experiments.

### 3.6. Profiling Community Composition

Two soil samples were used to isolate soil bacterial communities. Sample 1 was collected from a wet patch of orchard colonized by hornworts and shamrocks (San Pedro de la Viña, Zamora, Spain) while sample 2 was collected from a waste ground with cruciferous and grass plants (Leganés, Madrid, Spain). The soil samples were taken just behind the plant aerial parts (ca. 2-3 cm), cutting the small roots and including them in the soil sample. Aliquots of 10 g were homogenized using a mortar handle for 5 min in 30 mL PBS 1X. Samples were stirred with a magnet for 15 min/2500 rpm and then transfer to a 100 mL graduate cylinder, and let it settle for 10 min to allow sedimentation of large particles. To further eliminate soil clumps, 25 mL was recovered and spun down at 400 rpm/15 min. Twenty mL of supernatant was collected and centrifuged at 4000 rpm/15 min. The cellular pellet was washed in 20 mL PBS 1X and centrifuged at 4000 rpm/15 min. The sample was washed again with 2 mL PBS 1X and finally resuspended in 2 mL of the same buffer, obtaining samples S1 and S2 (final OD_600_ ~7.0). Aliquots of S1 and S2 were plated on MT-Cx solid media to screen the bacterial population. Plates were incubated 3 days at 30°C, and 30 bacterial colonies from both samples were selected by differences in size, colour, and morphology and streaked in the same media. 16S gene amplification was performed by colony PCR with primers 27F/1492R, and amplicons were sequenced with the same primers. 16S sequences were manually assembled with DNASTAR Lasergene software, and a homology search was done in the GenBank database using *blastn*. Samples S1 and S2 were also plated on MT-Km-Cx to examine the presence of Km^R^ bacteria in the soil samples.

### 3.7. Bulk Delivery of the Mini-RP4 Plasmid to the Extracted Soil Microbiota

To prepare the receptor samples for matings with the microbiota of the two soils under investigation, aliquots of S1 and S2 corresponding to 0.5 unit of OD_600_ (~ 70 *μ*L) were spun down at 11000 rpm/2 min and resuspended in 500 *μ*L 10 mM MgSO_4_. The donor strain *P. putida* EM42 *ΔpyrF Δ*K1-T6SS/pMATING*α*-msfGFP and the same strain with the control plasmid pSEVA221*α*-msfGFP were grown overnight in 10 mL of MT-Ura-Km. After measuring the optical density, culture aliquots of 0.5 unit of OD_600_ were centrifuged at 11000 rpm/2 min and resuspended in 500 *μ*L 10 mM MgSO4. Matings with the soil microbiota were prepared by mixing 0.5 mL of a donor strain with 0.5 mL of a soil sample, spinning down the cells at 11000/2 min, washing the cells with 1 mL of 10 mM MgSO_4_, and finally resuspending the cell pellet in 20 *μ*L of the same buffer. The mating samples were placed on top of a solid plate of MT-Ura and incubated 20 h at 30°C. Bacteria were recovered by scraping out the cellular patch with an inoculation loop and resuspending the sample in 1 mL of 10 mM MgSO_4_. Serial dilutions were plated on MT-Km-Cx, and plates were incubated 3 days at 30°C. Thirty fluorescent colonies were streaked on the same media several times to obtain pure cultures of each strain. Isolated strains were characterized by 16S amplification and sequencing as explained above. The presence of pMATING*α*-msfGFP was further analyzed by diagnostic PCR with primers L2-F1/Tra1-R2: all selected strains showed the expected amplification band of 0.5 Kb (Supplementary Figure S1).

### 3.8. Delivery of pMATING*α*-msfGFP Donors to Soil Microcosms

Natural samples were used to assess the ability of *P. putida* to transfer pMATING*α*-msfGFP to the soil bacterial community under environmental conditions. Samples were obtained from the same location selected to isolate sample 1. Two patches of soil and ground cover were extracted by digging around the selected area (25×25 cm, depth 15 cm). The samples were transferred to plant pots, watered with 200 mL of tap water, and placed in an open backyard. Donor cultures of *P. putida* EM42 *Δ*K1-T6SS *ΔpyrF* harbouring pMATING*α*-msfGFP or pSEVA221-msfGFP (control) were grown overnight in 400 mL of MT-Ura-Km. The cultures (DO_600_ ~1.0) were centrifuged at 4000 rpm/20 min, washed twice with 50 mL PBS 1X, and resuspended in 200 mL PBS 1X. The plant pots were inoculated with the donor cultures by evenly pouring the cellular suspensions over the ground cover of the soil. After inoculation, the flowerpots were watered every other day with 200 mL of tap water and the temperature was measured twice per day (7:00 am and 4:00 pm). Samples of soil were taken after inoculation at days 3, 7, 21, and 35, and 10 g was processed as described above to isolate the bacterial community. Dilutions were plated in the solid media MT-Cx, MT-Km-Cx, and MT-Km-Cx-Ura to quantify, respectively, the culturable soil bacteria, the environmental transconjugants, and the *P. putida* donors. Plates were incubated for 3 days at 30°C, and the number of colonies was counted. For environmental transconjugants and *P. putida* donors, plates were inspected under UV light and only Km^R^/GFP+ colonies were counted. At each sampling point, the number of donors and transconjugants was divided by the number of culturable bacteria in the soil sample to obtain the ratios donors/soil bacteria and transconjugants/soil bacteria. The numbers were normalized to 1E+9 soil bacteria. In addition, transconjugants at different sampling points were picked up and streaked several times in MT-Km-Cx to obtain pure cultures. 16S amplification/identification was performed on 15 isolates as explained in the previous section together with PCRs to check the presence of pMATING*α*-msfGFP with primers L2-F1/Tra1-R2.

## Data Availability

The details of the constructs and determination of transfer frequencies used to support the findings of this study are included within the main body of this article and the Supplementary Information file.

## Supplementary Materials

Supplementary Materials Supplementary Table S1: bacterial strains and plasmids used in this work. Supplementary Table S2: oligonucleotides used 1 in this study. Supplementary Figure S1: colony PCR of 1 transconjugant.
