# Supplementary material for: Propagation of Recombinant Genes through Complex Microbiomes with Synthetic Mini-RP4 Plasmid Vectors
Source: Biodes Res. 2022 Aug 2;2022:9850305. doi: 10.34133/2022/9850305 (PMC10521647; doi:10.34133/2022/9850305)
Supplement: Supplementary Materials — Supplementary Table S1: bacterial strains and plasmids used in this work. Supplementary Table S2: oligonucleotides used 1 in this study. Supplementary Figure S1: colony PCR of 1 transconjugant. [file 9850305.f1.pdf]

Supplementary Information to Aparicio *et al.*

Supplementary Table S1. Bacterial strains and plasmids used in this work.

Strain or plasmid	Relevant characteristics[(a)]	Reference or source
Escherichia coli		
CC118	Cloning host; Δ(*ara-leu*) *araD* Δ*lacX74 galE*, *galK phoA thiE1 rpsE*(Sp^R), *rpoB*(Rif^R), *argE*(Am), *recA1*	[1]
CC118λ*pir*	CC118, λ*pir* lysogen	[2]
HB101	Helper strain used for conjugation; F^-, $λ^-$, *hsdS20* (rB^- mB^-), *rec*A13 *leuB6*(Am), *araC14* Δ(*gpt-pro*A)*62*, *lacY1 galK*2(Oc), *xyl-5*, *mtl-1*, *thiE1*, *rpsL20* (Sm^R), *glnX44* (AS)	[3]
TransforMax™ EC100D™ *pir* +	F^- *mcr*A Δ(*mrr*-hsdRMS-*mcrBC*) (ϕ*80 lac*ZΔM15) Δ*lac*X74 *rec*A1 *endA1 araD139* Δ(*ara*,*leu*)*7697 gal*U *galK* $λ^-$ *rpsL nupG pir*+(DHFR).	Lucigen®
MC1061 DIAL EI Δ*dapA*::FRT-Km^R-FRT-Eps	DAP auxothoph, Km^R<br>F^- $λ^-$ Δ(*ara-leu*)7697 [*araD*139]B/r Δ(*codB*-*lacI*) 3 *galK16 galE15* e14$^-$ *mcrA0 relA1 rpsL150* (Sm^R) *spoT1 mcrB1 hsdR*2(r^-m^+) Δ*dapA*::FRT-Km^R-FRT Epsilon-His *pi-RepA* (medium-copy support for R6K, ColE2 origins of replication)	Tom Ellis Collection
MC1061 DIAL EI ΔdapA-Eps	DAP auxothoph, Km^S<br>F^- $λ^-$ Δ(*ara-leu*)7697 [*araD*139]B/r Δ(*codB*-*lacI*) 3 *galK16 galE15* e14$^-$ *mcrA0 relA1 rpsL150* (Sm^R) *spoT1 mcrB1 hsdR*2(r^-m^+) Δ*dapA* Epsilon-His *pi-RepA* (medium-copy support for R6K, ColE2 origins of replication)	This work
Pseudomonas putida		

EM42	KT2440 derivative; Δprophage1 Δprophage4 Δprophage3 Δprophage2 ΔTn7 Δ*endA-1* Δ*endA-2* Δ*hsdRMS* Δflagellum ΔTn*4652*	[4]
EM42 Δ*pyrF*	EM42 derivative; Δ*pyrF*	[5]
EM42 ΔK1-T6SS	EM42 derivative; ΔK1-T6SS	This work
EM42 Δ*pyrF* ΔK1-T6SS	EM42 derivative; Δ*pyrF* ΔK1-T6SS	This work
Saccharomyces cerevisiae		
CRY1-2	MATα, *ura3*, Δ*cyh2R*	Lab collection
B. subtillis		
BG214	*trpCE metA5 amyE1 ytsJ1 rsbV37 xre1 xkdA1* $att^{SPß}$ $att^{ICE\mathit{Bs1}}$	JC Alonso Collection
Plasmids		
pRK600	Helper plasmid used for conjugation; *oriV*(ColE1), RK2 (mob+ tra+); Cm^R	[6]
pSEVA222Sβ	Yeast shuttle vector; $oriV_{RK2}$; Km^R; cargo [*lacZ*α-pUC19/I-SceI]; gadget β (CEN6-ARS209-URA); *oriT*	[7]
pSEVA222Sβ-MATING	Yeast shuttle vector; $oriV_{RK2}$; Km^R; cargo [*lacZ*α-pUC19/I-SceI]; gadget β (CEN6-ARS209-URA); *oriT*; MATING module (Tra1- *xylS*-P_m -Tra2)	This work
pTRANS	Mini-Tn5-TRANS delivery plasmid; $oriV_{R6K}$; Ap^R; Km^R; CEN6-ARS209-URA3; TRANS module (Tra1- *xylS*-P_m -Tra2 Cores);	[7]
pSEVA221	Cloning vector; $oriV_{RK2}$; standard multiple cloning site; Km^R	[8]
pSEVA221α	Cloning vector; $oriV_{RK2}$; standard multiple cloning site; Km^R; gadget α (*hok-sok*)	[8]
pSEVA221α^{oriT}	$oriV_{RK2}$; standard multiple cloning site; Km^R; gadget α (*hok-sok*); SEVA *oriT* deleted	This work

pSEVA221α-msfGFP	*oriV*$_{RK2}$; Km^R; gadget α (*hok-sok*); P$_{EM7}$ →[msfGFP]	This work
pSEVA227-M-PEM7-msfGFP	*oriV*$_{RK2}$; Km^R; P$_{EM7}$ →[msfGFP]	[9]
pMATING	*oriV*$_{RK2}$; Km^R; MATING module (Tra1- *xylS*-P$_m$ - Tra2); *oriT*	This work
pMATINGα	*oriV*$_{RK2}$; Km^R; MATING module (Tra1- *xylS*-P$_m$ - Tra2); gadget α (*hok-sok*)	This work
pMATING2α	*oriV*$_{RK2}$; Km^R; MATING module (Tra1- *xylS*-P$_m$ - Tra2); gadget α (*hok-sok*); SEVA *oriT* deleted	This work
pMATINGα-msfGFP	*oriV*$_{RK2}$; Km^R; MATING module (Tra1- *xylS*-P$_m$ - Tra2); *oriT*; gadget α (*hok-sok*); P$_{EM7}$ →[msfGFP]	This work
pSEVA412S-3104-3110	*oriV*$_{R6K}$; cargo [*lacZ*α-pUC19/I-SceI]; Sm^R; TS1 (PP3104)-TS2 (PP3110)	[10]
pSEVA528S	I-SceI expressing plasmid; *oriV*$_{RK2}$ *xylS*-P$_m$→*I-SceI;* Tc^R	[10]
pFLP2	FLP recombinase expressing plasmid; oriV$_{ColE1}$-oriV$_{pRO1600}$; *sacB;* Ap^R; cI857-P$_L$→[FLP recombinase]	[11]
pSEVA2a2d1	*oriV*$_{RK2}$-*oriV*$_{pSM19035}$; Km^R$_{S. aureus}$; standard multiple cloning site	Laboratory collection
pSEVA2a2d1-MATING	*oriV*$_{RK2}$-*oriV*$_{pSM19035}$; Km^R$_{S. aureus}$; MATING module	This work
pC194	Cm$^{R\text{-}S. aureus}$	[12]
pBT233	*oriV*$_{pSM19035}$	[13]
pUB110	Km$^{R\text{-}S. aureus}$	[14]

[a] Antibiotic markers: Ap, ampicillin; Cm, chloramphenicol; Km, kanamycin; Gm, gentamicin; Rif, rifampicin; Sm, streptomycin; Tc, tetracycline

Supplementary Table S2. Oligonucleotides used in this study.

Name	Sequence (5’ → 3’)(a)	Usage/Source(b)
27F	AGAGTTTGATCCTGGCTCAG	Universal primers to amplify the bacterial 16S gene [15]
1492R	GGTTACCTTGTTACGACTT	
Tra1-R2	TCAAACAAGCCCAGCTAACG	To check the presence of the MATING module (*xylS*-Tra1 region, 0.5 Kb) [7]
L2-F1	TGCTATGAGCGACCAGATTG	
Gibson-PstI-msfGFP-F	cttaggagctacgaccatgcaactgaaaaaagcgctgcaTTGTTGACAATTAATCATCGGC	To amplify the P_{EM7}-msfGFP cassette and clone by Gibson Assembly in *PstI* site of pMATING (PstI site mutated in blue)
Gibson-PstI-msfGFP-F2	ttcgagctcggtacccggggatcctctagagtcgacctgcagTTGTTGACAATTAATCATCGGC	To amplify the P_{EM7}-msfGFP cassette and clone by Gibson Assembly in *PstI* site of pSEVA plasmids
Gibson-PstI-msfGFP-R	ttcccagtcacgacgcggccgcaagcttgcatgcctgcagTTATTTGTAGAGTTCATCCATGCC	To amplify the P_{EM7}-msfGFP cassette and clone by Gibson Assembly in *PstI* site of pSEVA plasmids
PP3104-EcoRI-F	aacgacggccagtatagggataacagggtaatctgaattcGTCAACCTGGATGCAGGC	To amplify TS1(PP3104)-TS2(PP3110) of K1-T6SS of *P. putida* KT2440 (1.0 Kb) [10]
PP3110-BamHI-R	ctagaagcttgcatgcctgcaggtcgactctagaggatccCGACGCAGACCCCGGAGC	
PP3106-F	CAGCCAACAGAGCGATGTAA	To amplify a 0.5 Kb fragment of PP3106 of *P. putida* KT2440 K1-T6SS [10]
PP3106-R	GCGTTGACTGCCACTGAGTA	
T1-Fw	GAGAGCGTTCACCGACAAAC	To amplify Ab^R-MCS region of pSEVAs and delete the *oriT* sequence
Del-oriT-Rv	cctgcatcgcggccggccgaccgcgGTCCAATTAATTATTAG	
Del-oriT-Fw2	ggccggccgcgatgcaggTGGCTGCTGAACCCCCAG	To amplify *ori* region of pSEVAs and delete the *oriT* sequence
T1-Rv	CTGGGCCTTTCGTTTTATCTG	
Km-check1	TCAGGTGCTGGAAGAATATC	To sequence oriT region in pSEVA221α
trfA-check1	CGATCACCTTCACGTTCTAC	To sequence oriT region in pSEVA221α
PS1	AGGGCGGCGGATTTGTCC	To sequence pSEVA-derived plasmids [8]
PS3	GAACGCTCGGTTGCCGC	
PS4	CCAGCCTCGCAGAGCAGG	
PS5	CCCTGCTTCGGGGTCATT	
PS6	GGACAAATCCGCCGCCCT	

(a) Restriction sites appear in red.

(b) A reference source only appears for primers designed in other works.

Supplementary Fig. S1. Colony PCR of trans-conjugants.

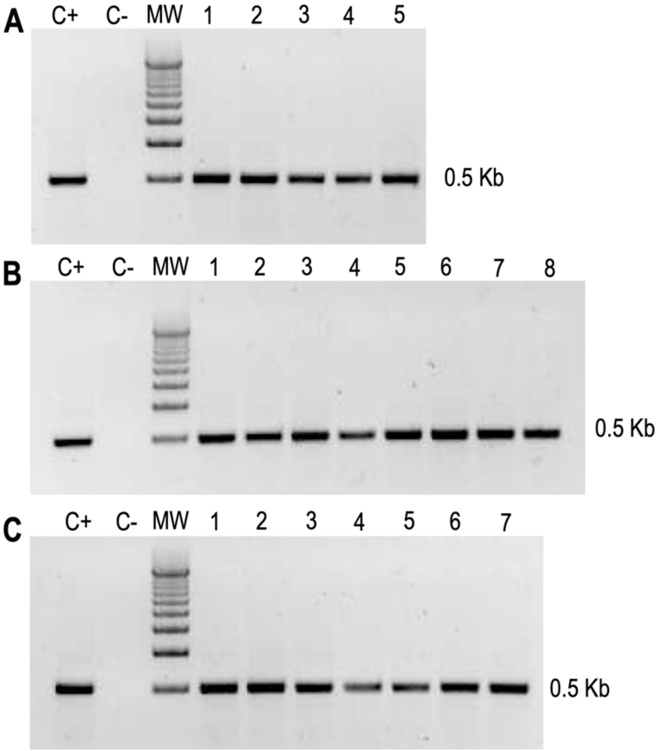

Primers L2-F1/ Tra1-R2 were used to verify the transfer of mini-RP4 derivatives to the recipient strains in the different experiments described in this work. Five to thirty trans-conjugants from each set of experiments were verified (see main text for details). In all cases the PCR showed the expected band corresponding to the amplified *xylS*-Tra1 region of the MATING module (0.5 Kb). Only representative amplifications are showed in this Figure, together with a positive control amplification of pMATING plasmid (C+) and a negative control (C-). **(A)** PCRs of trans-conjugants described in Fig. 2. Lanes 1 to 5 corresponds, respectively, to *E. coli* CC118/ pMATING, *P. putida* EM42/ pMATING, *P. putida* EM42 ΔK1-T6SS / pMATING, *B. subtillis*/ pSEVA2a2d1-MATING, *S. cerevisiae*/ pSEVA222Sβ-MATING. **(B)** PCRs of trans-conjugants described in Fig. 3. Lanes 1 to 8 corresponds, respectively, to the amplifications of the following strains harboring pMATINGα-msfGFP: *Novosphingobium sp., Pantoea sp., Buttiauxella sp., Aeromonas sp., Pseudomonas-1, Pseudomonas-2, Pseudomonas-3 and Enterobacter sp.* (C) PCRs of trans-conjugants described in Fig. 5 and 6. Lanes 1 to 6 corresponds to amplifications of receptors *E. coli*/ pMATINGα-msfGFP receiveing the plasmid from, respectively, *Novosphingobium sp., Pantoea sp., Buttiauxella sp., Aeromonas sp., Pseudomonas-1 and Pseudomonas-2.* Lane 7 corresponds to a receptor *E. coli*/ pMATING2α.

REFERENCES

[1] Grant, SG, Jessee, J, Bloom, FR, Hanahan, D. 1990 Differential plasmid rescue from transgenic mouse DNAs into Escherichia coli methylation-restriction mutants. Proc Natl Acad Sci U S A 87, 4645-4649.

[2] Herrero, M, de Lorenzo, V, Timmis, KN. 1990 Transposon vectors containing non-antibiotic resistance selection markers for cloning and stable chromosomal insertion of foreign genes in gram-negative bacteria. J Bacteriol 172, 6557-6567. (DOI:10.1128/jb.172.11.6557-6567.1990).

[3] Boyer, HW, Roulland-Dussoix, D. 1969 A complementation analysis of the restriction and modification of DNA in Escherichia coli. J Mol Biol 41, 459-472.

[4] Martinez-Garcia, E, Nikel, PI, Aparicio, T, de Lorenzo, V. 2014 Pseudomonas 2.0: genetic upgrading of P. putida KT2440 as an enhanced host for heterologous gene expression. Microb Cell Fact 13, 159. (DOI:10.1186/s12934-014-0159-3).

[5] Aparicio, T, Jensen, SI, Nielsen, AT, de Lorenzo, V, Martinez-Garcia, E. 2016 The Ssr protein (T1E_1405) from Pseudomonas putida DOT-T1E enables oligonucleotide-based recombineering in platform strain P. putida EM42. Biotechnol J 11, 1309-1319. (DOI:10.1002/biot.201600317).

[6] Kessler, B, de Lorenzo, V, Timmis, KN. 1992 A general system to integrate lacZ fusions into the chromosomes of gram-negative eubacteria: regulation of the Pm promoter of the TOL plasmid studied with all controlling elements in monocopy. Mol Gen Genet 233, 293-301.

[7] Silbert, J, Lorenzo, V, Aparicio, T. 2021 Refactoring the Conjugation Machinery of Promiscuous Plasmid RP4 into a Device for Conversion of Gram-Negative Isolates to Hfr Strains. ACS Synth Biol 10, 690-697. (DOI:10.1021/acssynbio.0c00611).

[8] Silva-Rocha, R, Martinez-Garcia, E, Calles, B, Chavarria, M, Arce-Rodriguez, A, de Las Heras, A, Paez-Espino, AD, Durante-Rodriguez, G, Kim, J, Nikel, PI, et al. 2013 The Standard European Vector Architecture (SEVA): a coherent platform for the analysis and deployment of complex prokaryotic phenotypes. Nucleic Acids Res 41, D666-675. (DOI:10.1093/nar/gks1119).

[9] Tas, H, Grozinger, L, Goni-Moreno, A, de Lorenzo, V. 2021 Automated design and implementation of a NOR gate in Pseudomonas putida. Synth Biol (Oxf) 6, ysab024. (DOI:10.1093/synbio/ysab024).

[10] Aparicio, T, de Lorenzo, V, Martínez-García, E. 2017 Broadening the SEVA Plasmid Repertoire to Facilitate Genomic Editing of Gram-Negative Bacteria. In Hydrocarbon and Lipid Microbiology Protocols: Genetic, Genomic and System Analyses of Pure Cultures (eds. TJ McGenity, KN Timmis & B Nogales), pp. 9-27. Berlin, Heidelberg, Springer Berlin Heidelberg.

[11] Hoang, TT, Karkhoff-Schweizer, RR, Kutchma, AJ, Schweizer, HP. 1998 A broad-host-range Flp-FRT recombination system for site-specific excision of chromosomally-located DNA sequences: application for isolation of unmarked Pseudomonas aeruginosa mutants. Gene 212, 77-86. (DOI:10.1016/s0378-1119(98)00130-9).

[12] Horinouchi, S, Weisblum, B. 1982 Nucleotide sequence and functional map of pC194, a plasmid that specifies inducible chloramphenicol resistance. J Bacteriol 150, 815-825. (DOI:10.1128/jb.150.2.815-825.1982).

[13] Ceglowski, P, Boitsov, A, Chai, S, Alonso, JC. 1993 Analysis of the stabilization system of pSM19035-derived plasmid pBT233 in Bacillus subtilis. Gene 136, 1-12. (DOI:10.1016/0378-1119(93)90441-5).

[14] McKenzie, T, Hoshino, T, Tanaka, T, Sueoka, N. 1986 The nucleotide sequence of pUB110: some salient features in relation to replication and its regulation. Plasmid 15, 93-103. (DOI:10.1016/0147-619x(86)90046-6).

[15] Weisburg, WG, Barns, SM, Pelletier, DA, Lane, DJ. 1991 16S ribosomal DNA amplification for phylogenetic study. J Bacteriol 173, 697-703. (DOI:10.1128/jb.173.2.697-703.1991).
